# Myo-inositol improves developmental competence and reduces oxidative stress in porcine parthenogenetic embryos

**DOI:** 10.3389/fvets.2024.1475329

**Published:** 2024-12-13

**Authors:** Ali Jawad, Dongjin Oh, Hyerin Choi, Mirae Kim, Jaehyung Ham, Byoung Chol Oh, Joohyeong Lee, Sang-Hwan Hyun

**Affiliations:** ^1^Veterinary Medical Center and College of Veterinary Medicine, Laboratory of Veterinary Embryology and Biotechnology (VETEMBIO), Chungbuk National University, Cheongju, Republic of Korea; ^2^Institute of Stem Cell and Regenerative Medicine (ISCRM), Chungbuk National University, Cheongju, Republic of Korea; ^3^Department of Plastic and Reconstructive Surgery, Johns Hopkins University School of Medicine, Baltimore, MD, United States; ^4^Department of Companion Animal Industry, College of Healthcare and Biotechnology, Semyung University, Jecheon, Republic of Korea; ^5^Vet-ICT Convergence Education and Research Center (VICERC), Chungbuk National University, Cheongju, Republic of Korea; ^6^Chungbuk National University Hospital, Cheongju, Republic of Korea

**Keywords:** myo-inositol, embryos, mitochondria, parthenogenesis, oxidative stress

## Abstract

**Objective:**

Myo-inositol (Myo-Ins), the most abundant form of inositol, is an antioxidant and plays a crucial role in the development and reproduction of mammals and humans. However, information elucidating the role of Myo-Ins in porcine embryonic development after parthenogenetic activation (PA) is still lacking. Therefore, we investigated the effect of Myo-Ins on porcine embryos and its underlying mechanisms.

**Methods:**

In this study, various concentrations of Myo-Ins (0, 5, 10, and 20 mM) were added to the porcine zygotic medium (PZM3) during the *in vitro* culture (IVC) of porcine embryos. Several characteristics were evaluated, including cleavage rate, blastocyst formation rate, intracellular glutathione (GSH) and reactive oxygen species (ROS) levels in 4–5 cell stage embryos, total cell number, apoptotic rate in blastocysts, mitochondrial membrane potential (MMP), mitochondrial quantity, mitochondrial stress in the blastocysts, and gene expression for antioxidant and mitochondrial function markers. Additionally, the immunofluorescence of *HO-1* was assessed.

**Results:**

The results showed that Myo-Ins at concentrations of 10 and 20 mM significantly increased the blastocyst formation rate compared to the control group. Embryos supplemented with 20 mM Myo-Ins exhibited higher GSH levels and lower ROS levels than those in the control group. Myo-Ins supplementation also decreased the rate of apoptosis and the apoptotic index in the treatment groups. Additionally, embryos supplemented with 20 mM Myo-Ins showed increased mitochondrial membrane potential (MMP), greater mitochondrial quantity, and reduced oxidative stress in the mitochondria. Interestingly, the expression levels of genes related to mitochondrial function and the nuclear erythroid factor 2-related factor (*NRF2*) pathway were elevated in the Myo-Ins treated groups. Furthermore, immunofluorescence results indicated that 20 mM Myo-Ins significantly increased *HO-1* expression in blastocysts compared to the control group.

**Conclusion:**

In conclusion, 20 mM Myo-Ins supplementation enhanced blastocyst development and improved mitochondrial function by regulating apoptosis, reducing oxidative stress, and activating the *NRF2* pathway.

## 1 Introduction

*In vitro* embryo production (IVP) is an essential technique for early-stage porcine embryos that provides sufficient embryos for biomedical technology uses, such as genetic modification and cloning, compared to *in vivo* embryos. Consequently, the creation of porcine models for biomedical research is an important step in the IVP embryos with high implantation potential (1, 2). The quality of embryos during IVP directly affects the subsequent implantation of embryos and the early development of the fetus. In IVP, the easiest method to evaluate pre-implantation embryos is the blastocyst formation rate; nonetheless, this technique has not significantly improved during the past 20 years. The *in vitro* culture (IVC) system’s inability to accurately mimic the natural *in vivo* microenvironment of embryo development is an obstacle to making significant advancements in the assessment of blastocyst formation rate, particularly in porcine embryos. This is mainly because pig embryos are extremely susceptible to reactive oxygen species (ROS) impeding their growth (3, 4). Thus, it is crucial to optimize embryo culture systems to improve the competency and quality of early embryonic development.

Excessive ROS levels result in oxidative stress, which ultimately affects embryonic development (5). ROS can result from the surrounding culture environment or the embryo’s metabolism, and cause protein denaturation (6), lipid peroxidation (7), DNA damage (8), and mitochondrial damage (9), which, in turn, results in the inhibition of embryo development (10). Enhancing the efficiency and quality of porcine embryo production is a viable strategy for impeding the generation of excessive ROS. Previous studies have demonstrated that the addition of antioxidants, such as melatonin (11), cysteine (12), linoleic acid (13), laminarin (14), and resveratrol (15), diminishes the oxidative stress of embryos. Nevertheless, the existing IVC conditions fail to mimic the *in vivo* environment. Consequently, understanding the mechanisms of antioxidative damage and investigating potent antioxidant substances for incorporation into culture media are effective approaches for enhancing the *in vitro* developmental capabilities of embryos (10).

Inositol is a crucial component of structural lipids. Phosphatidylinositol, a key element in cellular membranes, especially mitochondrial membranes, plays a pivotal role in the maintenance and function of mitochondria (16). Myo-inositol (Myo-Ins), the predominant form of inositol in nature, actively participates in cytogenesis, cell morphogenesis, lipid synthesis, cell membrane formation, and cell growth. Myo-Ins is recognized as a precursor of secondary messengers in cell signaling systems and is therefore involved in the regulation of intracellular calcium concentrations (17, 18). Consequently, it plays a crucial role in regulating cardiac function, increasing insulin sensitivity, influencing metabolic changes, and remarkably impacting reproductive processes (19–22). Myo-Ins present in body fluids, particularly the follicular fluid, plays a crucial role in generating essential intracellular signals. Additionally, it is vital for follicle maturation of follicles and serves as an indicator of high oocyte quality (23–25).

Research on farming animals has indicated that Myo-Ins plays a role in mammalian preimplantation development, as the supplementation of culture media with Myo-Ins increases blastocysts formation, expansion, and hatching in rabbits and bovines (26, 27), ultimately supporting the development of healthy animals (26). In addition, it has been shown that Myo-Ins exhibits antioxidant properties and mitigates oxidative stress (28, 29). Previous studies have reported that Myo-Ins plays a protective role against oxidative stress by activating the *NRF2/KEAP1* signaling pathway (30, 31). However, there is a lack of evidence elucidating the association between Myo-Ins and the *NRF2/KEAP1* signaling pathway, specifically in porcine *in vitro* embryonic development. Therefore, the objective of this study was to explore porcine *in vitro* embryonic development and elucidate the underlying mechanisms by incorporating optimal concentrations of Myo-Ins into the IVC medium. This involved the assessment of blastocyst quality, monitoring of intracellular levels of ROS and glutathione (GSH), and evaluation of mitochondrial quantity and membrane potential. Furthermore, we aimed to determine the correlation between Myo-Ins and the *NRF2/KEAP1* signaling pathway during porcine early embryogenesis.

## 2 Materials and methods

### 2.1 Chemical and reagents

All the chemicals and reagents were purchased from Sigma-Aldrich (St.Louis, MO, United States). MitoSOX Red mitochondrial superoxide indicator and JC-1 were obtained from Invitrogen by Thermo Fisher Scientific (Carlsbad, CA, United States). *HO-1/HMOX-1* primary antibody was purchased from Proteintech (10701-1-AP, Proteintech, Illinois, USA).

### 2.2 Oocyte collection and *in vitro* maturation (IVM)

Porcine ovaries were procured from a local abattoir and promptly transported to the laboratory within 3 h in a 0.9% (v/v) NaCl solution maintained at 37–39°C. Upon arrival, the ovaries were subjected to two washes with a 0.9% (v/v) NaCl solution. Medium-sized follicles (3–7 mm in diameter) were selected for the retrieval of cumulus-oocyte complexes (COCs) using an 18G needle and a 10 mL disposable syringe (32). Subsequently, the collected complexes were transferred to 15 mL conical tubes. COCs exhibiting evenly granulated cytoplasm and compact cumulus cell layers were selected, and 50–60 COCs were cultured in each well of a four-well dish (Nunc, Roskilde, Denmark) containing 500 μL of IVM medium and not covered with mineral oil. The IVM medium consisted of TCM199 (Invitrogen Corporation, Carlsbad, CA, United States) supplemented with 0.91 mM sodium pyruvate, 0.6 mM cysteine, 75 ug/mL kanamycin, 10 ng/mL epidermal growth factor, 10% (v/v) porcine follicular fluid, and 1 μg/mL insulin. During the initial 22 h of IVM, the COCs were incubated with 10 IU/mL equine chorionic gonadotropin (eCG) and 10 IU/mL human chorionic gonadotropin (hCG), followed by a subsequent 20 h incubation without these hormones. All IVM procedures were conducted in a humidified incubator (Astec, Fukuoka, Japan) maintained at 39°C with a 5% CO₂ atmosphere.

### 2.3 Parthenogenetic activation (PA) and *in vitro* culture (IVC) of porcine embryos

PA was conducted in accordance with a previously published protocol (33). Initially, metaphase II stage (MII) oocytes were collected following IVM and treated with 0.1% hyaluronidase for 1 min to facilitate the removal of cumulus cells through gentle mechanical pipetting. Subsequently, the MII oocytes were rinsed twice with an activation solution comprising 280 mM mannitol, 0.01 mM CaCl₂ and 0.05 mM MgCl₂ before undergoing activation in 2 mL of the same solution. Activation was achieved through the administration of two direct electrical pulses at 120 V/mm for 60 μs, delivered via a cell fusion generator (LF101; NepaGene, Chiba, Japan). Subsequently, the activated oocytes were cultured in PZM3 containing cytochalasin B within a humidified environment of 5% CO_2_, 5% O_2_, and 95% N_2_ for 4 h (34). All embryos were subjected to electrical activation and underwent three washes with IVC medium droplets (containing 10 oocytes per 30 μL) fully covered with mineral oil. PA was initiated on day 0, with embryo transferring to fresh droplets on days 2 (48 h) and 4 (96 h) of development. The cleavage rates of the embryos were analyzed on the second day and categorized into five groups based on the number of cells: 1, 2–3, 4–5, 6–8 cells, and fragmented embryos. On the seventh day, embryonic development was quantitatively assessed by determining blastocyst formation rates across three groups categorized by blastocyst morphology: early, expanded, and hatched. To quantify cell numbers in blastocysts, all blastocysts displaying a blastocoel were stained with 10 μg/mL Hoescht-33342 for 10 min. Following staining, each blastocyst was mounted on a glass slide in a drop of 100% and examined under an epifluorescence microscope (TE 300; Nikon, Tokyo, Japan) at 200 x magnification. The cell numbers were manually counted, and blastocysts with a blastocoel and at least 20 cells were classified as blastocysts (34–36). Myo-Ins supplementation was introduced into the IVC media at concentrations of 0 (control), 5, 10, and 20 mM, without any prior research on its use. The optimal concentration for Myo-Ins supplementation was determined through analysis, as depicted in Supplementary Figure S1 and Supplementary Table S1.

### 2.4 Measurement of intracellular GSH and ROS levels

As previously described (37) intracellular GSH and ROS levels were assessed following established procedures. Embryos at the 4–5 cell stage were selected from each experimental group on day 2 for analysis. Intracellular GSH (indicated by blue fluorescence) and ROS (indicated by green fluorescence) levels within the embryo cytoplasm were quantified utilizing CellTracker Blue CMF_2_HC (Invitrogen) and H_2_DCFDA (Invitrogen), respectively. The embryos were stained by incubating them in a solution of TLH-PVA medium containing either 10 μM CMF_2_HC or H_2_DCFDA for 30 and 10 min, respectively. Following this, the samples were washed three times with TLH-PVA before being transferred to 8 μL droplets of TLH-PVA. The levels of glutathione (GSH) and ROS were quantified using a fluorescence microscope (TE300; Nikon, Tokyo, Japan) equipped with ultraviolet filters (370 nm for GSH and 460 nm for ROS). The fluorescence intensity of each embryo was then assessed using Image J software and normalized against that of the control group.

### 2.5 Terminal deoxynucleotidyl transferase-mediated dUTP nick end labeling assay (TUNEL assay)

The number of apoptotic cells in the blastocysts stained with TUNEL was determined using an *in-situ* cell death detection kit as previously described (38). On the seventh day, Myo-Ins-treated and control group blastocysts were washed three times in 0.1% PBS-PVA (PVS). The blastocysts were then fixed in 4% paraformaldehyde (PFA) in PBS at 25°C (room temperature (RT)) for 30 min. In the subsequent stage, the blastocysts underwent two washes in a solution containing 0.1% PVS, in conjunction with 0.1% Tween 20 and 0.01% Triton X-100 (v/v). Subsequently, the blastocysts were treated with 0.3% Triton X-100 in PBS for 1 h at 37°C in order to facilitate permeabilization. Subsequently, the TUNEL assay was conducted utilizing fluorescein-conjugated deoxy uridine triphosphate (dUTP) and terminal deoxynucleotidyl transferase (obtained from Roche, Mannheim, Germany) for 90 min at 37°C. Subsequently, the blastocysts were subjected to two further washes in 0.1% PVS, after which they were counterstained with 5 μg/mL Hoechst-33342 for 10 min at RT in order to visualize the nuclei.

### 2.6 Assay of mitochondrial membrane potential (∆Ψm)

Blastocysts on day 7 were cultured in PZM 3 supplemented with 2.5 μM 5,5′,6,6′-tetrachloro-1,1′,3,3′-tetraethyl-imidacarbocyanine iodide (JC-1) (Cat # T3168, Invitrogen, Eugene, OR, USA) for 30 min at 38.5°C in 5% CO_2_. Membrane potential was determined by calculating the ratio of red fluorescence, which corresponds to activated mitochondria (J-aggregates), to green fluorescence, which corresponds to less activated mitochondria (J-monomers) (39). Images were captured using an epifluorescence microscope (TE300; Nikon, Tokyo, Japan), and Image J software was used to quantify the relative fluorescence intensities.

### 2.7 Colocalization assay of the mitochondria and mitochondrial ROS

To examine the colocalization of the mitochondria and mitochondrial ROS, blastocysts were incubated with 500 nM MitoTracker Red CMXRos (Cat #M7512, Invitrogen, Eugene, OR, USA) and 0.4 μM MitoSOX red (Cat # 2201584, Invitrogen, Oregon, USA) at 38.5°C for 30 min (39, 40). The fluorescence signals were visualized using an epifluorescence microscopy (TE300; Nikon, Tokyo, Japan) and the relative fluorescence intensities were quantified using the Image J software.

### 2.8 Quantitative reverse transcription-polymerase chain reaction

Blastocysts (*n* = 20/group) were collected from the control and Myo-Ins treated groups and washed with DPBS before being stored in 1.5 mL microcentrifuge tubes (SPL Life Sciences, Co., Ltd., Pocheon, Gyeonggi-do, Republic of Korea) and placed at −80°C until analysis. For the extraction of total RNA, TRIzol reagent (TaKaRa Bio, Inc., Otsu, Shiga, Japan) was used. Following the manufacturer’s protocol, cDNA was synthesized using a 5× reverse transcription master mix (Elpis Bio, Inc., Chungcheongnam-do, Daejeon, Republic of Korea). For qRT-PCR, the synthesized cDNA (0.5 μg/μL) was mixed with 2× SYBR Premix Ex Taq (TaKaRa Bio Inc.) and 10 pmol of specific primers (Macrogen, Inc., Seoul, Republic of Korea). The primers used in this study are listed in Supplementary Table S2. A CFX96 Touch real-time PCR detection system (Bio-Rad, Hercules, CA, United States) was used for the qRT-PCR analysis. The reactions were initiated by pre-denaturation at 95°C for 5 min, followed by 40 cycles of denaturation at 95°C for 15 s, annealing at 57°C for 15 s, and extension at 72°C for 30 s. Data were collected during the extension phase of each cycle, and the relative quantification (R) value was calculated using the following equation: *R* = 2-^[ΔCtsample − ΔCtcontrol]^ (41). The expression levels of genes were normalized to *RN18S* as a control.

### 2.9 Immunofluorescence assay

The immunofluorescence technique was employed in accordance with the methodology described by Yoon et al. (42) with certain modifications. In brief, 4% PFA in PBS was employed to fix the blastocysts for 30 min at RT. Subsequently, the blastocysts were permeabilized in 0.5% Triton X-100 at RT for 1 h, after which they were washed twice with 0.1% PVS. The Image-iT™ FX Signal Enhancer (Invitrogen, Carlsbad, CA, United States) was employed to treat the blastocysts for a period of 30 min at RT. Subsequently, they were incubated in PBS containing 3% BSA and 0.05% Tween 20 for 1 h and 30 min at RT. The blastocysts were incubated overnight at 4°C with a rabbit anti-*HO-1/HMOX-1* primary antibody (10701-1-AP, 1:100 dilution in blocking buffer, Proteintech, Illinois, USA). On the following day, the blastocysts were washed three times with 0.1% Tween 20 and 0.01% Triton X-100 in 0.1% PVS (TTVS) at RT for 5 min. They were then incubated with the appropriate secondary antibody, goat anti-rabbit IgG (H + L) Alexa Fluor 488 (A11029, 1:200; Invitrogen Corporation, Carlsbad, CA, USA), for 2 h at RT. Following three washes in TTVS, the blastocysts were counterstained with Hoechst-33342 for 10 min and mounted on glass slides in an anti-fade mounting medium (Molecular Probes, Inc., Eugene, OR, USA). The stained blastocysts were analyzed using an epifluorescence microscope (TE 300; Nikon) equipped with UV filters. The ImageJ software was employed to quantify the fluorescence intensity of the stained blastocysts.

### 2.10 Experimental design

In experiment 1, a total of 954 embryos were used across three replicates to assess the effects of different concentrations of Myo-Ins (0, 5, 10, 20, 40, and 80 mM) on the *in vitro* development of porcine PA embryos. The embryos were cultured in IVC medium supplemented with the specified concentrations of Myo-Ins throughout the entire IVC period. Developmental competence was evaluated by measuring cleavage rate, blastocyst formation rate, and total cell number in blastocysts. Based on the results, high concentrations of Myo-Ins (40 and 80 mM) decreased the blastocyst formation rate, suggesting that these levels may negatively impact embryo development. Therefore, lower concentrations (0, 5, 10, and 20 mM) were selected for further experiments to optimize the *in vitro* development of porcine PA embryos. In experiment 2, 352 total embryos were used in three biological replicates to assess various concentrations of Myo-Ins (0, 5, 10, and 20 mM). We aimed to investigate whether Myo-Ins can reduce oxidative stress in early 4-cell staged PA embryos by monitoring intracellular GSH and ROS levels. In experiment 3, a total of 168 blastocysts were used across three independent replicates to evaluate the effect of different concentrations of Myo-Ins (0, 5, 10, and 20 mM) on apoptosis. The number of TUNEL-positive cells and cell number were measured to assess apoptosis levels in the blastocysts. In experiment 4, a total of 280 blastocysts were used in at least three replicates to investigate mRNA expression levels of antioxidant and mitochondrial related genes in porcine PA blastocysts. In experiment 5, a total of 281 blastocysts were used across three biological replicates to evaluate the (∆Ψm) in porcine blastocysts after PA with different concentrations of Myo-Ins (0, 5, 10, and 20 mM). In experiment 6, a total of 144 blastocysts were used across three biological replicates to assess mitochondrial distribution in porcine blastocysts after PA with different concentrations of Myo-Ins (0, 5, 10, and 20 mM). In experiment 7, a total of 167 blastocysts were used across three replicates to assess mitochondrial reactive oxygen species (ROS) levels in porcine blastocysts after PA with different concentrations of Myo-Ins (0, 5, 10, and 20 mM). In experiment 8, a total of 140 blastocysts were used across three replicates to investigate the effect of various concentrations of Myo-Ins (0, 5, 10, and 20 mM) on HO-1 protein levels after immunofluorescence assay.

### 2.11 Statistical analysis

Statistical analysis was performed using SPSS (version 12.0; SPSS, IBM, Armonk, NY, United States) and GraphPad Prism (GraphPad Software, San Diego, CA, United States). All the experiments were performed at least three times unless stated otherwise. Data were presented as the mean ± SEM. Percentage data (cleavage and blastocysts formation rates) and average data (intracellular GSH and ROS in 4–5 cell stage embryos, TUNEL assay in blastocysts, mitochondrial membrane potential, Mito tracker, MitoSoX, and relative gene expression levels) were analyzed using one-way analysis of variance. Statistical significance was set at *p* < 0.05.

## 3 Results

### 3.1 Myo-ins enhances development of porcine PA embryos

**Figure 1:**
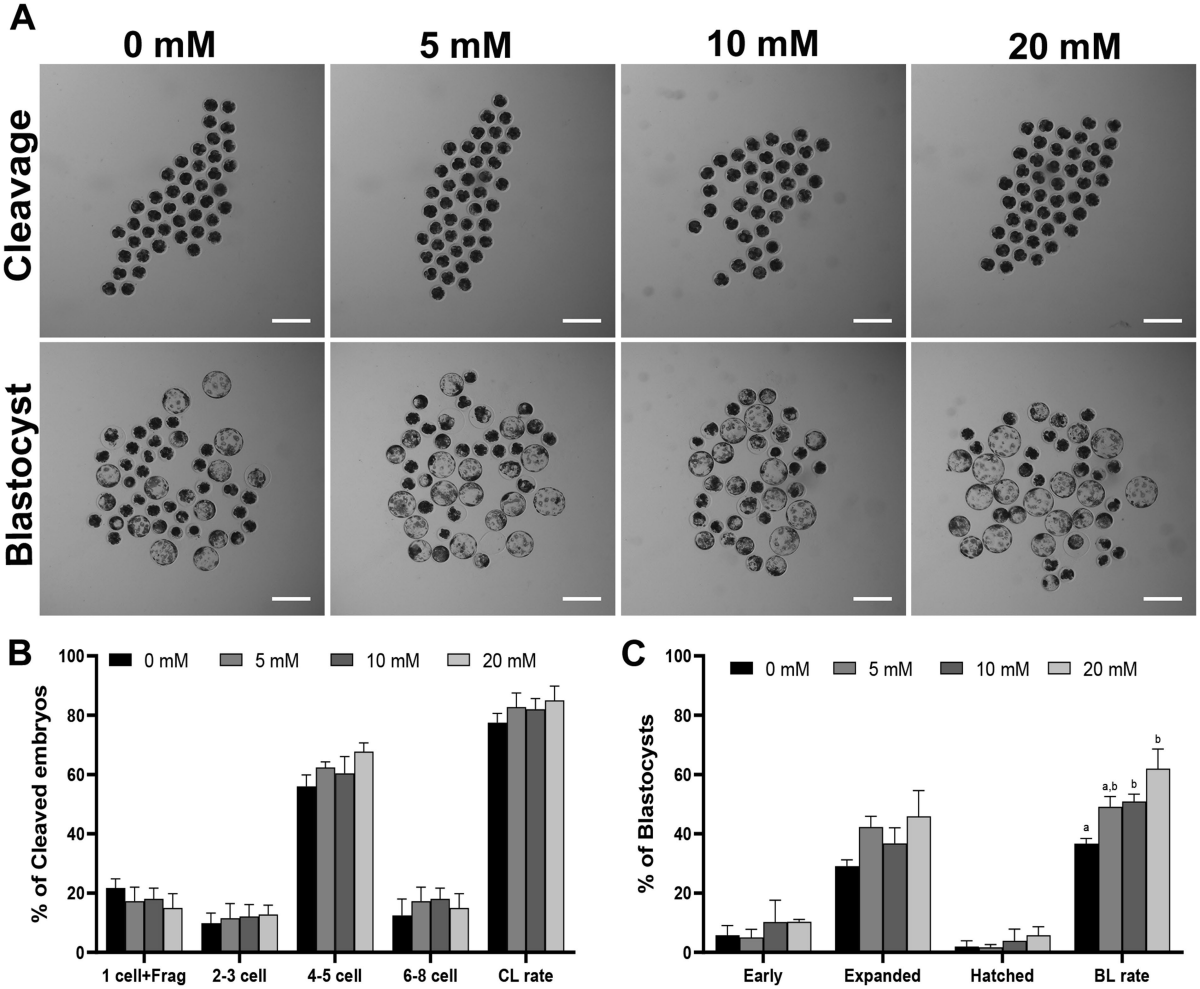
**(A)** Effect of Myo-Ins treatment on the blastocysts formation patterns of PA embryos. Scale bar = 300 μm. The cleavage pattern **(B)** and blastocyst formation **(C)** rate of PA embryos. Within each endpoint, the bars with different letters (a, b) indicate significant differences (*p* < 0.05) at various Myo-Ins concentrations. Frag, fragmentation; CL, cleavage; BL, blastocyst. The cleavage and blastocyst formation rates were evaluated on Day 2 and 7 after PA, respectively. For all the graphs, the values represent the mean ± SEM. The experiment was replicated at least three times.

The optimal concentration of Myo-Ins was investigated by adding different concentrations (0, 5, 10, 20, 40, and 80 mM) during IVC for porcine embryonic development following PA, as shown in Supplementary Figure S1 and Supplementary Table S1. Considering these findings, we selected the 5, 10 and 20 mM Myo-Ins treatment groups for this experiment. On day 2, the embryonic cleavage rate was evaluated in all the Myo-Ins treatment groups (82.7% ± 4.7, 82.0% ± 3.6, 85.0% ± 4.8%) compared with the control group (77.5% ± 3.1%) (Table 1) (Figures 1A,B). Myo-Ins treatment did not show any differences in the cleavage rate when compared with the control group. The blastocyst formation rates were found to be significantly increased (*p* < 0.05) in the 10 (50.9% ± 2.5%) and 20 mM (61.9% ± 6.7%) Myo-Ins treated groups compared to the control group (36.7% ± 1.7%) (Figures 1A,C). The supplementation of Myo-Ins at concentrations of 5-(37.0% ± 0.7%), 10-(38.7% ± 2.6%) and 20-(40.2% ± 3.2%) did not result in a significant increase in the average total cell number of blastocysts in comparison to the control group (40.5% ± 2.2%) (Table 1).

**Table 1:** Effect of Myo-Ins treatment during *in vitro* culture (IVC) for 7 days on embryonic development after parthenogenetic activation (PA).

Myo-Ins concentration (mM)	No. of embryos cultured, N	No. (%) of embryos developed to		Total cell number in blastocysts
		≥ 2-cell	Blastocyst	
0	114	88 (77.5 ± 3.1)	42 (36.7 ± 1.7) ^a^	40.5 ± 2.2
5	115	95 (82.7 ± 4.7)	57 (49.1 ± 3.5) ^a,b^	37.0 ± 0.7
10	106	87 (82.0 ± 3.6)	54 (50.9 ± 2.5) ^b^	38.7 ± 2.6
20	118	100 (85.0 ± 4.8)	72 (61.9 ± 6.7) ^b^	40.2 ± 3.2

N: Three times replicated. ^a, b^ Values with different superscripts within a column differ significantly (*p* < 0.05).

### 3.2 Myo-ins modulates intracellular GSH and ROS levels in PA cleaved embryos

**Figure 2:**
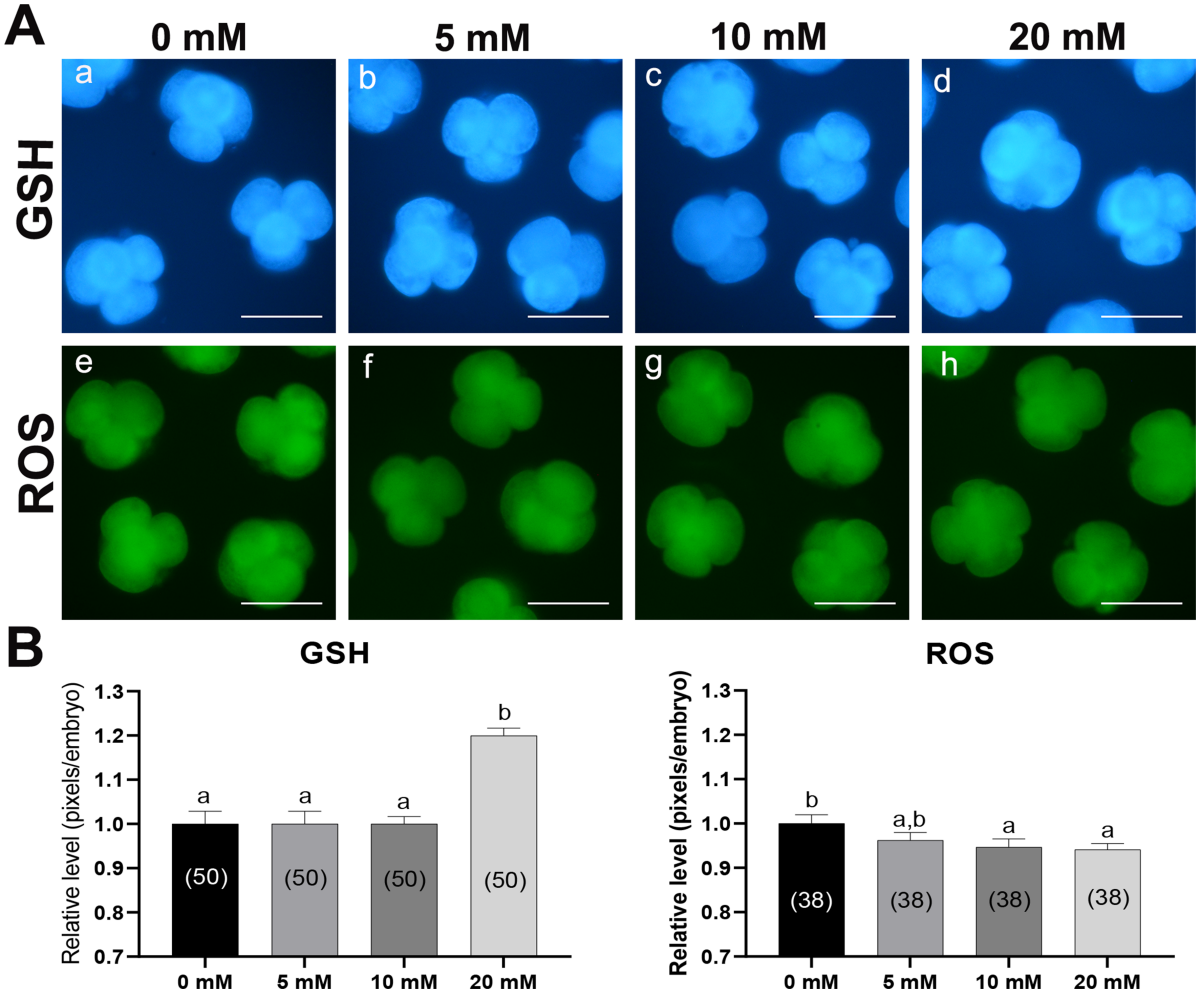
Epifluorescence photomicrographs and quantitative analysis of PA-derived 4-cell embryos with Myo-Ins supplementation during IVC for 2 days. **(A)** Representative epifluorescence photomicrographs of embryos stained with Cell Tracker Blue (a–d) and H_2_DCFDA (e–h), used to detect intracellular levels of glutathione (GSH) and reactive oxygen species (ROS), respectively. Scale bar = 100 μm. **(B)** The relative levels of intracellular GSH and ROS levels within the *in vitro* cultured porcine embryos treated with Myo-Ins during IVC. The number of embryos examined in each experimental group is indicated in parentheses. Bars with different letters (a, b) within each endpoint represent significant differences between groups (*p* < 0.05). Data are presented as mean ± SEM. The experiment was replicated three times. Scale bar = 100 μm.

To investigate the antioxidative effect of Myo-Ins, we analyzed the intracellular GSH and ROS levels in 4–5 cell stage embryos cultured with Myo-Ins-supplemented medium on day 2 after PA (Figure 2). The 20 mM Myo-Ins group showed significantly higher (*p* < 0.05) intracellular GSH levels than those in the control group. Furthermore, embryos in the 10 and 20 mM treated groups displayed significantly lower (*p* < 0.05) intracellular ROS levels compared to than those in the control group.

### 3.3 Myo-ins regulates apoptosis in porcine PA embryos

**Figure 3:**
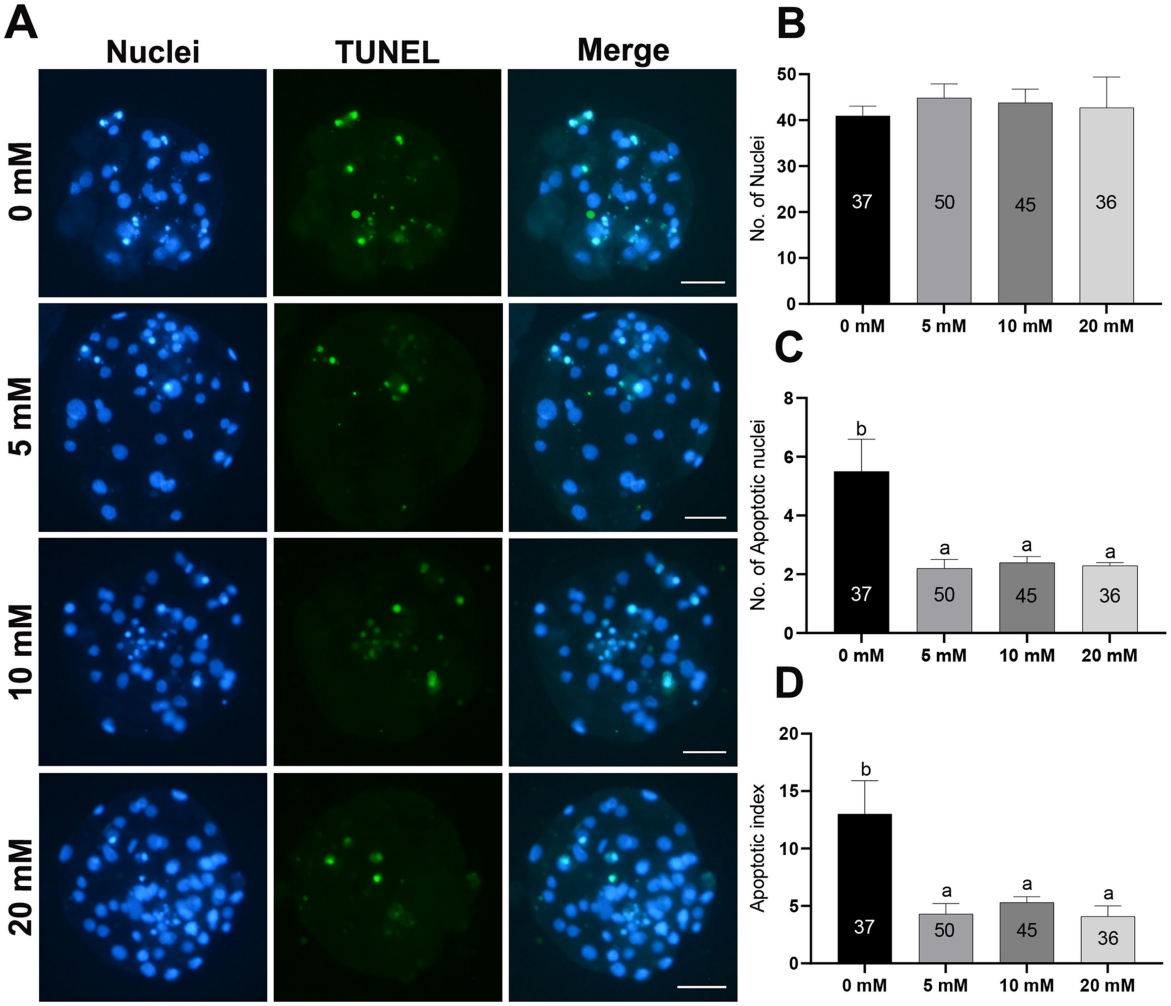
Total cell number and apoptotic nuclei in PA-derived blastocysts treated with different concentrations of Myo-Ins during IVC for 7 days. **(A)** TUNEL assay of porcine PA blastocysts in the control and Myo-Ins treated groups. The blastocysts were stained green for TUNEL and the nuclei were stained blue with Hoechst (33342). Scale bar = 50 μm. **(B–D)** Quantification of the total and apoptotic cell numbers, and apoptotic index in the indicated groups. The number of embryos examined in each experimental group is shown in parentheses. Within each endpoint, the bars with different letters (a,b) indicate significant differences (*p* < 0.05) for each group. For all the graphs, the values represent the mean ± SEM. The experiment was replicated at least three times.

To evaluate the quality of porcine PA blastocyst, the incidence of apoptosis and total nuclei were counted. Myo-Ins supplementation during IVC did not affect the total number of nuclei compared to the control group (Figures 3A,B). Nonetheless, the number of apoptotic nuclei and the apoptotic index significantly decreased (*p* < 0.05) in the Myo-Ins-treated groups compared to those in the control group (Figures 3A,C,D).

### 3.4 Myo-ins modifies gene expression in porcine PA embryos

**Figure 4:**
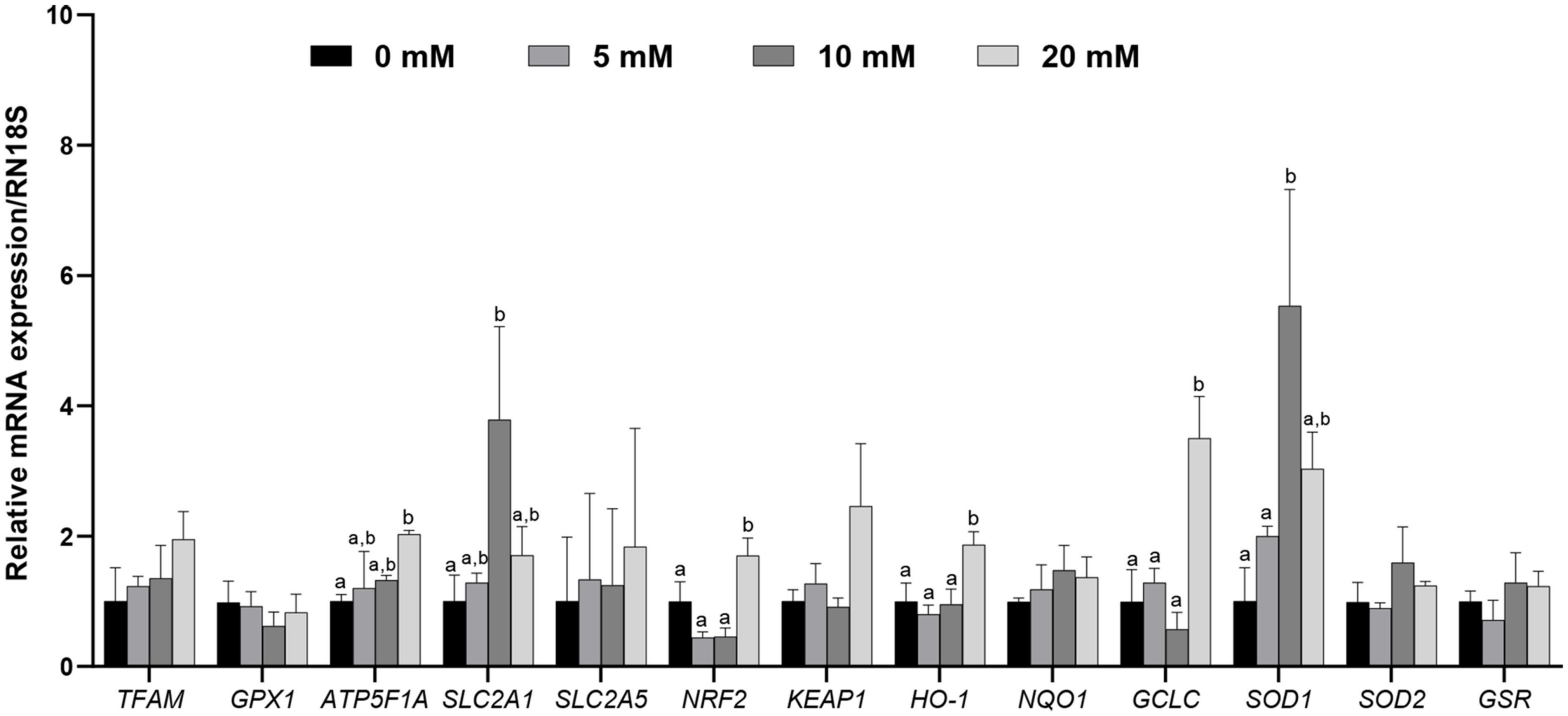
Relative mRNA expression levels of genes associated with mitochondria, including mitochondrial transcription factor A (*TFAM*), glutathione peroxidase 1 (*GPX1*), ATP synthase (*ATP5F1A*), solute carrier family 2 member 1 (*SLC2A1*), and solute carrier family 2 member 5 (*SLC2A5*), along with genes associated with antioxidant signaling, including nuclear erythroid factor 2 related genes (*NRF2*), Kelch like associated protein (*KEAP1*), Heme oxygenase 1 (*HO-1*), quinine oxidoreductase 1 (*NQO1*), glutamate-cysteine ligase catalytic subunit (*GCLC*), superoxide dismutase 1, 2 (*SOD1, SOD2*), and glutathione-disulfide reductase (*GSR*), were analyzed in porcine blastocysts treated with Myo-Ins at concentrations of 5, 10, and 20 mM, with data normalized to the *RN18S* gene and different letters (a, b) indicating significant differences between the groups, and all values representing mean ± SEM from experiments replicated three or four times.

To investigate whether Myo-Ins supplementation during IVC after PA affects the expression levels of mitochondrial function-related genes and antioxidant pathway genes such as *NRF2/HO-1*, blastocysts from each group were analyzed (Figure 4). Myo-Ins significantly increased (*p* < 0.05) the expression of mitochondrial function-related genes, such as solute carrier family 2 member 1 (*SLC2A1*) and ATP synthase (*ATP5F1A*), in the 10 and 20 mM groups compared to the control group (Figure 4). Additionally, the mRNA transcript levels of *NRF2*, *HO-1*, and *GCLC* were significantly higher (*p* < 0.05) in the 20 mM Myo-Ins group than in the control group. *SOD1* transcript levels were significantly higher in the 10 mM Myo-Ins-treated group than in the control group. However, no significant differences were observed in the other mitochondrial-related genes or antioxidant genes when compared to the control group (Figure 4).

### 3.5 Myo-ins improves mitochondrial function in porcine PA embryos

**Figure 5:**
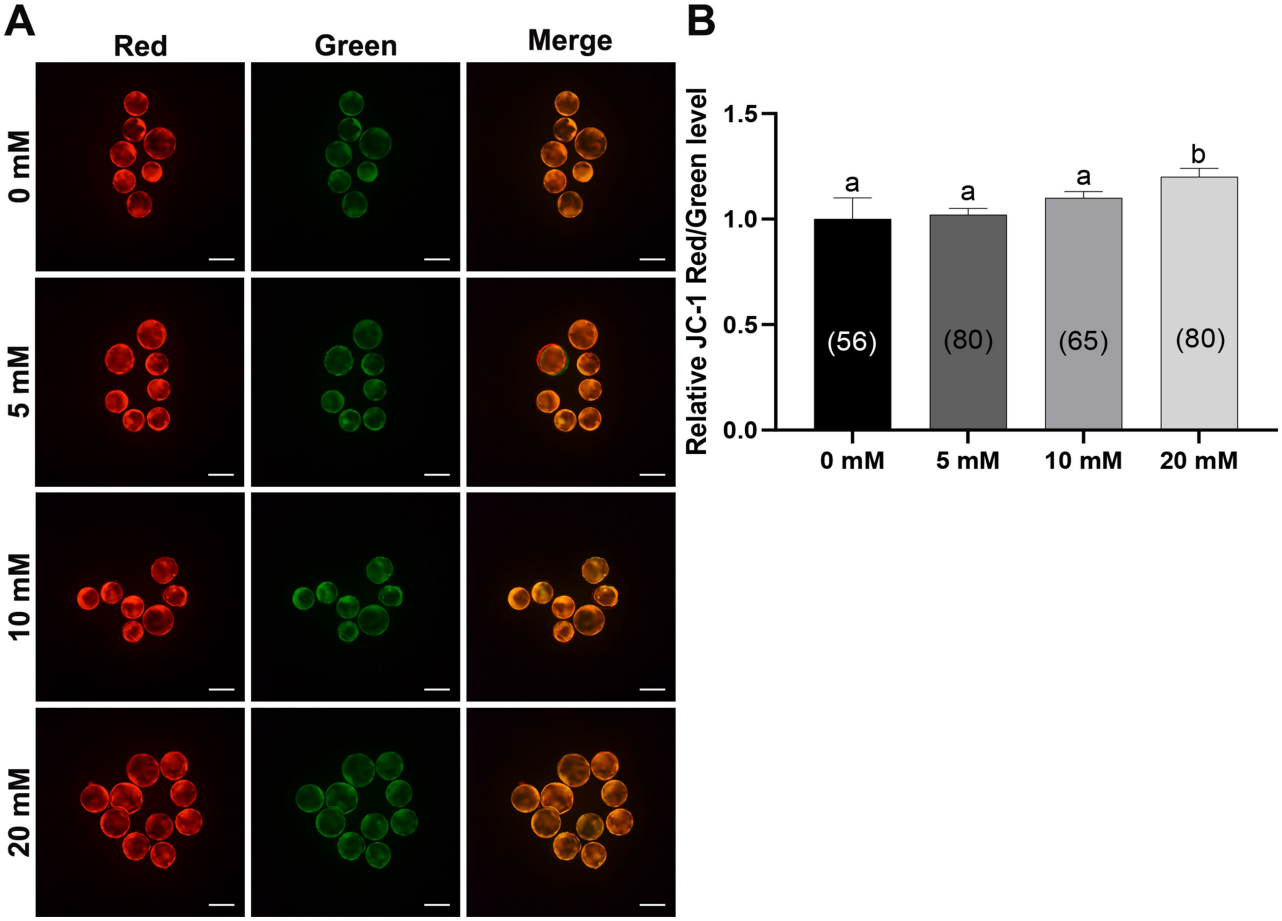
Myo-Ins prevented mitochondrial dysfunction in porcine blastocysts. **(A)** Representative fluorescence images of JC-1 staining in blastocysts. Scale bar = 200 μm. **(B)** Quantification of the ratio of fluorescence intensity (red/green) of JC-1 in blastocysts. The number of the blastocysts is shown in parentheses. Data are expressed as the mean ± SEM. Within each endpoint, bars with different letters (a, b) indicate significant differences (*p* < 0.05). The experiment was replicated at least four times.

**Figure 6:**
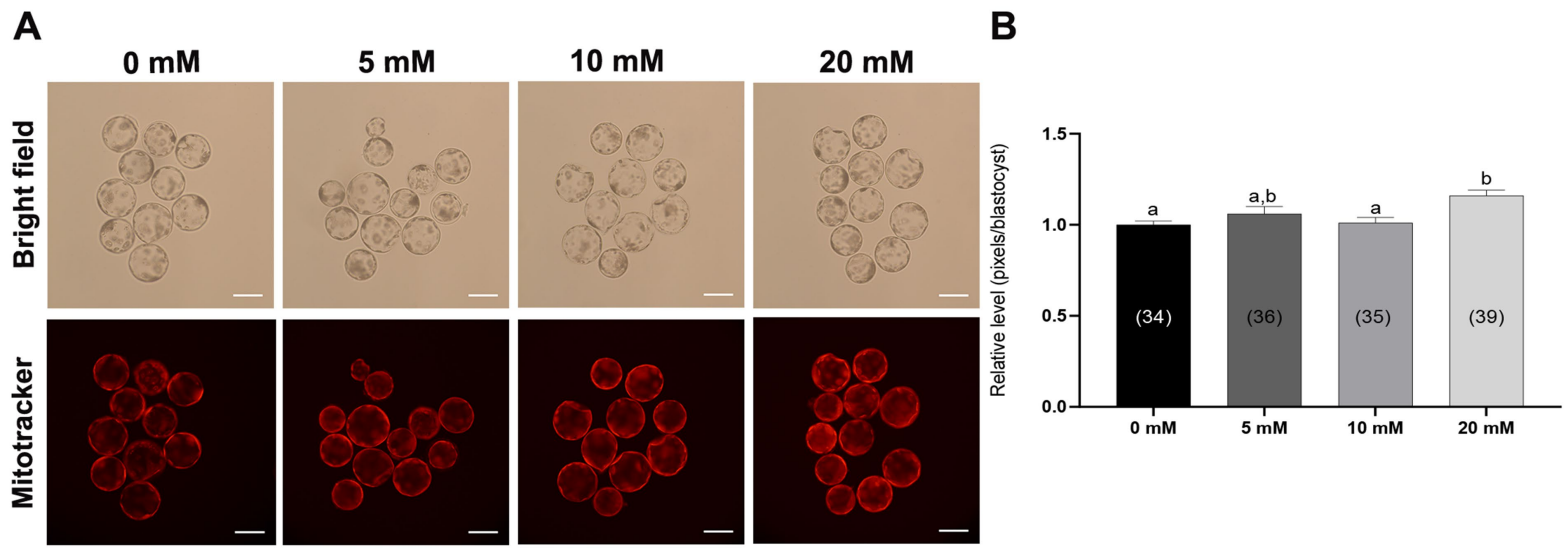
Effect of Myo-Ins on mitochondrial function in porcine PA embryos. **(A)** Representative fluorescent images of MitoTracker Deep Red staining of the Myo-Ins treated groups (5, 10, and 20 mM) and control group. Scale bar = 100 μm. **(B)** Relative MitoTracker fluorescence intensities in the blastocysts of the Myo-Ins treated groups and control group. The number of blastocysts is indicated in parentheses. Within each endpoint, bars with different letters (a, b) indicate significant differences (*p* < 0.05) at various Myo-Ins concentrations. For all the graphs, the values represent the mean ± SEM. The experiment was replicated three times.

**Figure 7:**
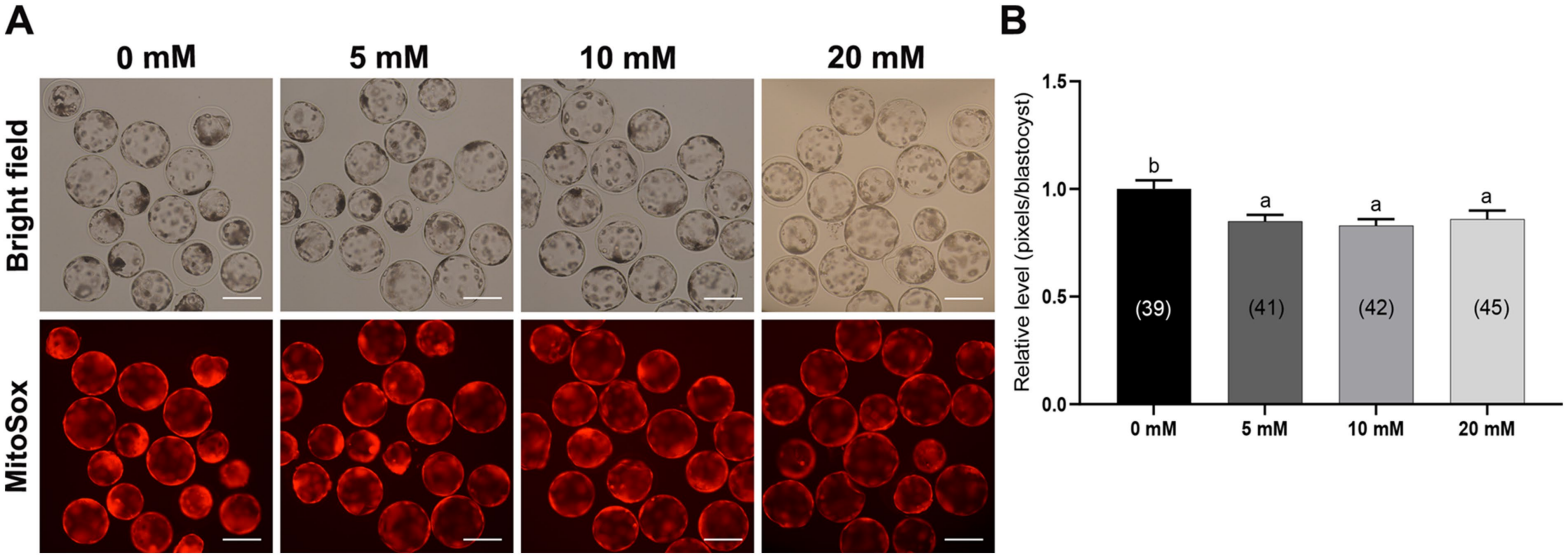
Effect of Myo-Ins on mitochondrial ROS in porcine PA embryos. **(A)** Representative fluorescent images of MitoSox staining of the Myo-Ins treated groups (5, 10, and 20 mM) and control group. Scale bar = 200 μm. **(B)** Relative MitoSox fluorescence intensities in the blastocysts of the Myo-Ins treated groups and control group. The number of blastocysts is indicated in parentheses. Within each endpoint, bars with different letters (a, b) indicate significant differences (*p* < 0.05) at various Myo-Ins concentrations. For all the graphs, the values represent the mean ± SEM. The experiment was replicated three times.

Mitochondria are the main organelles responsible for ROS production, and any malfunction regarding them may compromise embryonic development (43, 44). Therefore, Myo-Ins supplementation in the IVC medium was assessed in three ways: mitochondrial membrane potential (MMP), mitochondrial distribution (Mitotracker), and mitochondrial ROS (MitoSox). Myo-Ins at 20 mM significantly improved (*p* < 0.05) mitochondrial dysfunction by enhancing MMP (Figures 5A,B). Moreover, the mitochondrial quantity also significantly increased (*p* < 0.05) in the 20 mM Myo-Ins-treated group compared to that in the control group (Figures 6A,B). In addition, mitochondrial oxidative stress was reduced (*p* < 0.05) in the Myo-Ins-treated groups compared to that in the control group (Figures 7A,B).

### 3.6 Myo-ins activates *HO-1* expression in porcine PA embryos

**Figure 8:**
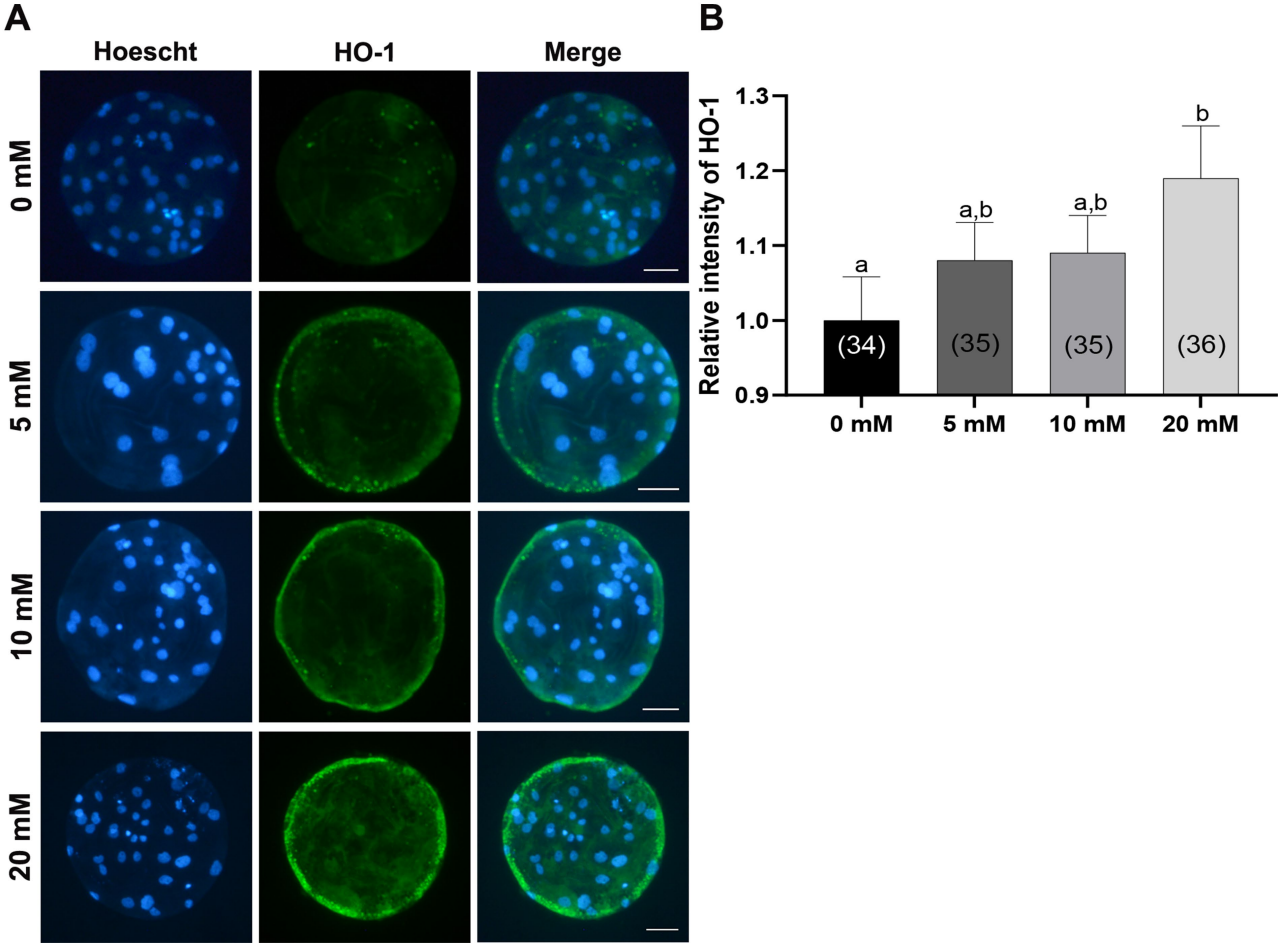
Effects of Myo-Ins treatment during *in vitro* culture (IVC) for 7 days on the heme oxygenase-1 (*HO-1*) expression of parthenote embryos. **(A)** Immunofluorescence images (200x) of porcine parthenote blastocysts labeled with *HO-1* (green) and Hoechst 33342 (total nuclei, blue) following *in vitro* culture (IVC) for 7 days. Scale bar = 50 μm. **(B)** The relative intensity of *HO-1* was quantified in both the control and Myo-Ins treatment groups. The number of embryos analyzed in each experimental group is indicated in brackets. Within each endpoint, bars with different letters (a, b) indicate significant differences (*p* < 0.05) at different concentrations of Myo-Ins. The graph displays the mean ± SEM values. The experiment was replicated three times.

Previous study reported the antioxidant role of Myo-Ins against oxidative stress in boar sperm in which *NRF2* gene expression was significantly increased compared to that in the control group. However, downstream genes of the *NRF2* pathway have not been discussed before in porcine IVC (30). Therefore, to investigate whether Myo-Ins supplementation during IVC stimulates the *NRF2/HO-1* pathway at the porcine blastocyst stage, we analyzed the expression of the *HO-1* protein using immunofluorescence. The blastocysts treated with 20 mM Myo-Ins showed a significant increase (*p* < 0.05) in the level of *HO-1* protein compared to that in the control group (Figures 8A,B).

## 4 Discussion

This study aimed to examine the effects of Myo-Ins supplementation on porcine PA embryos during IVC. During IVC, embryos are more susceptible to oxidative stress because of the differences between the *in vivo* and *in vitro* environments. Oxidative stress induced by ROS can cause various impairments in developmental parameters, such as decreased blastocyst formation rate and cell number (45). Antioxidant supplementation can improve embryonic developmental competence by preventing the excessive accumulation of ROS. Therefore, the effect of Myo-Ins on porcine parthenogenetic preimplantation embryos was investigated. We also investigated the optimal concentration of Myo-Ins in porcine parthenogenetic embryos during IVC. The addition of Myo-Ins significantly increased the blastocyst formation rate and GSH levels, and decreased the ROS levels in 4–5 cell stage embryos. Additionally, apoptosis in blastocysts was downregulated by the inclusion of Myo-Ins. Moreover, Myo-Ins supplementation improves mitochondrial dysfunction in blastocysts by reducing oxidative stress and increasing mitochondrial distribution, resulting in increased MMP levels. In addition, supplementation with Myo-Ins significantly increased the expression of genes related to the *NRF2/HO-1* pathway, mitochondrial function markers, and *HO-1* proteins in the blastocysts.

Enhancing embryo quality is crucial for developing effective animal models for biomedical research. Nevertheless, unlike the controlled environment of the oviduct, the *in vitro* setting exposes embryos to various stress-inducing factors, leading to an uneven distribution of antioxidants and free radicals in the cytoplasm (46). Thus, minimizing oxidative stress is important to improve the developmental competence and quality of *in vitro-*produced porcine embryos. In the present study, we confirmed that supplementation with Myo-Ins during IVC enhanced the developmental competence of porcine parthenogenetic (PA-derived) embryos. Of the various concentrations (0, 5, 10, and 20 mM) of Myo-Ins added, only the inclusion of 10 and 20 mM Myo-Ins in the IVC medium significantly increased the blastocyst formation rate. Therefore, the optimal Myo-Ins concentration for porcine IVC was identified as 20 mM which is similar to previous study in which the addition of 20 mM Myo-Ins increased the developmental competence of mouse oocytes (47).

The idea that a regulated redox system is crucial for normal embryonic development is supported by the observation that dysregulation of redox equilibrium severely affects normal embryonic development (48). During IVC, the embryos are prone to damage caused by ROS-induced oxidative stress. These ROS function as secondary messengers and regulate essential transcription factors associated with oxidative processes (49). Moreover, GSH levels are crucial during the oxidative stress process in porcine or rodent embryos, as GSH plays a vital role in scavenging ROS in damaged cells and is associated with cell proliferation events during embryonic development (50–52). A previous study has revealed the antioxidant properties of Myo-Ins in murine MII oocytes. It effectively reduced ROS levels and increased GSH levels after a preincubation period of 4 h (47). In this study, the elevated intracellular GSH levels and reduced ROS levels in the embryos treated with Myo-Ins on day 2 indicate that the *in vitro* system’s uncontrolled balance between pro-oxidative and antioxidative stresses, which can inhibit embryonic development, might be able to be regulated by Myo-Ins supplementation.

Apoptosis is a programmed cell death process that occurs regularly to maintain a balance between cell formation and cell death. It is essential for homeostasis and regulates numerous genes. However, excessive apoptosis can lead to the oocyte degeneration and early embryo death and can also disrupt normal blastocyst formation (53). In this study, apoptosis and the apoptotic index were significantly downregulated in the Myo-Ins treated groups compared to those in the control group. In contrast, an increase in apoptosis can indicate inadequate *in vitro* conditions for oocytes (54). Therefore, Myo-Ins inclusion prevented apoptosis in the blastocysts; however, further research is needed to investigate the mechanisms of action of Myo-Ins against apoptosis in porcine blastocysts.

Mitochondria are vital organelles that play a crucial role in embryonic development and with excessive ROS levels disrupt mitochondrial function (43, 44). Disruption of mitochondrial function impairs embryonic development and causes abnormal autophagy and apoptosis, leading to death (55, 56). MMP is a commonly used indicator of mitochondrial function. However, excessive ROS accumulation can hinder mitochondrial biogenesis, which involves the synthesis of new mtDNA, mitochondrial division, and membrane formation (57). A study conducted on mouse oocytes found that preincubation with 20 mM Myo-Ins for 4 h and 8 h improved MMP (47). In the present study, mitochondrial dysfunction was improved by the enhancement of MMP and the distribution of mitochondrial quantity within Myo-Ins-treated blastocysts. Furthermore, Myo-Ins treatment decreased the mitochondrial ROS levels, specifically superoxide levels, in porcine blastocysts. These results are consistent with those of a previous study conducted in mice, in which pre-incubation with 20 mM Myo-Ins improved MMP and mitochondrial distribution in their oocytes (47). However, previous research was only conducted on mouse oocytes, and this is the first report of Myo-Ins supplementation in porcine blastocysts during IVC, in which mitochondrial function improved. Based on these results, we speculated that Myo-Ins enhances mitochondrial function in porcine blastocysts by regulating oxidative stress.

A previous study revealed that Myo-Ins supplementation during the liquid preservation of boar sperm improved oxidative stress, increased the activity of the nuclear factor (erythroid-derived 2) like 2 (*NRF2*)-regulated antioxidant pathway and acted as a ROS scavenger (30). *NRF2* is a well-known transcription factor involved in antioxidant protein expression (58). Under normal conditions, *NRF2* remains inactive because it is tethered to its negative regulator, Kelch-like associated protein 1 (*KEAP1*), which is located in the cytoplasm. However, during oxidative stress, it detaches from *KEAP1* and translocates to the nucleus, where it binds to ARE (antioxidant response elements) (59). Subsequently, genes are activated by the expression of antioxidant enzymes such as *HO-1*and *SOD*, as well as enzymes responsible for the formation of GSH, are expressed (59). In this study, 20 mM Myo-Ins treatment significantly upregulated *NRF2* gene expression and the expression of downstream genes such as *HO-1* and *GCLC*. However, *SOD1* upregulation was observed with 10 mM the Myo-Ins, illustrating the antioxidative role of Myo-Ins via the *NRF2/HO-1* pathway in porcine blastocysts. These results were further verified by immunofluorescence staining of the *HO-1* protein in blastocysts; 20 mM significantly increased *HO-1* expression in the blastocysts. Further studies are required to investigate the mechanisms underlying the antioxidant role of Myo-Ins in blastocysts via the *NRF2/HO-1* pathway.

The mitochondria are of significant importance in the generation of ATP during oocyte and embryonic development (60). This study found that blastocysts supplemented with Myo-Ins had higher mtDNA copy numbers and different mRNA expression levels of mitochondrial transcription factor A (*TFAM*) than the control group. May-Panloup et al. reported that mtDNA copy number may increase due to the elevated levels of *TFAM* transcripts, which rise simultaneously with mtDNA replication during bovine embryogenesis (61). However, this study illustrates that higher mtDNA copy numbers in porcine Myo-Ins-supplemented blastocysts may improve blastocyst quality with elevated mRNA expression levels of *TFAM* which is similar to the results of a previous study in bovines (61). Furthermore, in the blastocysts treated with Myo-Ins, there was a change in energy production via glucose metabolism, which is crucial for adequate mitochondrial function. Blastocysts supplemented with Myo-Ins showed elevated expression levels of *SLC2A1* and *SLC2A5*. These genes are responsible for active glucose and fructose transport across the plasma membrane, indicating the redirection of energy substrates towards anaerobic glycolysis (62). In oocytes, mitochondria play a crucial role in fertilization and embryonic developmental competence. They produce ATP through the expression of ATP synthase (*ATP5A1*) and regulate ROS via the oxidative stress marker glutathione peroxidase (*GPX1*), which is mediated by ROS (62). In this study, *ATP5F1A* was upregulated in the 20 mM Myo-Ins group compared to that in the control group, which showed high ATP production in the blastocysts. Moreover, the oxidative stress marker *GPX1* expression was slightly reduced with 10 and 20 mM Myo-Ins and significantly decreased in the 5 mM Myo-Ins treated group, indicating that a low concentration of Myo-Ins may be beneficial in lowering *GPX1* in porcine blastocysts. These results suggest that Myo-Ins may improve mitochondrial functions. However, further studies are required to assess these mitochondrial functional marker genes in porcine blastocysts to understand the underlying mechanisms.

## 5 Conclusion

In conclusion, the results of this study indicated that Myo-Ins supplementation in porcine PA embryos during IVC was highly efficient, highlighting the role of Myo-Ins in enhancing blastocyst formation rate and, decreasing apoptosis, and oxidative stress in 4–5 cell stage embryos. Moreover, Myo-Ins supplementation regulated mitochondrial dysfunction by increasing the mitochondrial distribution and MMP and reducing mitochondrial ROS. Furthermore, Myo-Ins also upregulated the *NRF2/HO-1* related pathway genes and mitochondrial function marker genes. *HO-1* protein expression was upregulated in blastocysts, highlighting the defense mechanism of Myo-Ins against oxidative stress. To the best of our knowledge, this was the first study to investigate the role of Myo-Ins in oxidative stress and mitochondrial dysfunction in porcine PA embryos. Overall, these findings offerred detailed and novel insights into the antioxidative, and anti-apoptotic protection provided by Myo-Ins in *in vitro* models.

## Data Availability

The datasets presented in this study can be found in online repositories. The names of the repository/repositories and accession number(s) can be found in the article/Supplementary material.

## Supplementary material

SUPPLEMENTARY FIGURE S1 Effect of various concentrations of Myo-Ins supplementation during in vitro culture (IVC) on embryonic development after parthenogenetic activation (PA). **(A)** Representative morphologies of porcine blastocysts from each group 7 days after PA. Scale bar = 300 µm. **(B)** Effect of Myo-Ins supplementation during IVC on the cleavage pattern of PA embryos at day 2. **(C)** Effect of Myo-Ins supplementation during IVC on the percentage of PA embryos that developed to the blastocyst stage at day 7. For all graphs, the value represents the mean ± SEM. Within each end point, bars with different letters (a and b) are significantly (*p* < 0.05) different. Statistical significance was determined by one-way ANOVA. 1 cell + fragmentation embryos; 2-3 cells; 4-5 cells; 6-8 cells; CL, cleavage; BL, blastocyst. The experiment was replicated three times.

SUPPLEMENTARY Table S1 Effect of Myo-Ins treatment during in vitro culture (IVC) for seven days on embryonic development after parthenogenetic activation (PA).
