# Supplementary figures and images for: Myo-inositol improves developmental competence and reduces oxidative stress in porcine parthenogenetic embryos
Source: Front Vet Sci. 2024 Dec 13;11:1475329. doi: 10.3389/fvets.2024.1475329 (PMC11672211; doi:10.3389/fvets.2024.1475329)

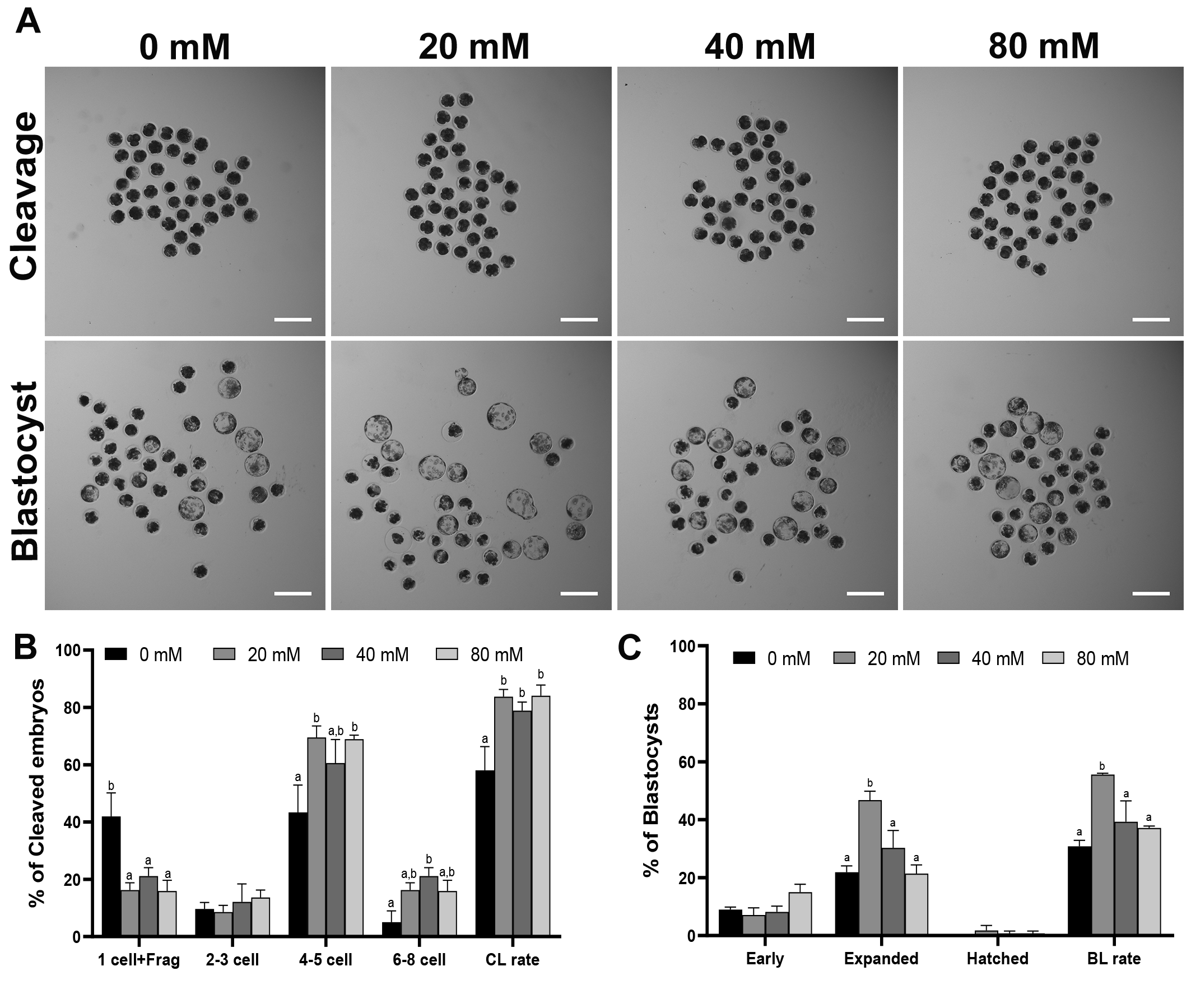

Supplement: SUPPLEMENTARY FIGURE S1 — Effect of various concentrations of Myo-Ins supplementation during in vitro culture (IVC) on embryonic development after parthenogenetic activation (PA). (A) Representative morphologies of porcine blastocysts from each group 7 days after PA. Scale bar = 300 µm. (B) Effect of Myo-Ins supplementation during IVC on the cleavage pattern of PA embryos at day 2. (C) Effect of Myo-Ins supplementation during IVC on the percentage of PA embryos that developed to the blastocyst stage at day 7. For all graphs, the value represents the mean ± SEM. Within each end point, bars with different letters (a and b) are significantly (p < 0.05) different. Statistical significance was determined by one-way ANOVA. 1 cell + fragmentation embryos; 2-3 cells; 4-5 cells; 6-8 cells; CL, cleavage; BL, blastocyst. The experiment was replicated three times. [file Image_1.tif]
