# Supplementary material for: Myo-inositol improves developmental competence and reduces oxidative stress in porcine parthenogenetic embryos
Source: Front Vet Sci. 2024 Dec 13;11:1475329. doi: 10.3389/fvets.2024.1475329 (PMC11672211; doi:10.3389/fvets.2024.1475329)
Supplement: SUPPLEMENTARY Table S1 — Effect of Myo-Ins treatment during in vitro culture (IVC) for seven days on embryonic development after parthenogenetic activation (PA). [file Table_1.docx]

**Supplementary Table 1.** Effect of Myo-Ins treatment during *in vitro* culture (IVC) for seven days on embryonic development after parthenogenetic activation (PA)

| **Myo-Ins concentration**  **(mM)** | **No. of embryos cultured, N** | | **No. of embryos developed to** | | |
| --- | --- | --- | --- | --- | --- |
|  |  |  | **≥ 2Cell** | **Blastocyst** | |
| 0 | 123 | 72 (58.1 ± 8.2) ^a^ | | 38 (30.8 ± 2.1) ^a^ |  |
| 20 | 124 | 104 (83.7 ± 2.5) ^b^ | | 69 (55.6 ± 0.5) ^b^ |  |
| 40 | 125 | 99 (78.9 ± 2.9) ^b^ | | 49 (39.3 ± 7.2) ^a^ |  |
| 80 | 129 | 109 (84.1 ± 3.8) ^b^ | | 48 (37.2 ± 0.7) ^a^ |  |

| **mRNA** | **Primer sequences** | | **Product size (bp)** | **GenBank accession number** |
| --- | --- | --- | --- | --- |
| ***RN18S*** | F: 5′-CGCGGTTCTATTTTGTTGGT-3′ | R: 5′-AGTCGGCATCGTTTATGGTC-3′ | 219 | NR_046261 |
| ***TFAM*** | F: 5′-CAGACTGGCAGGTGTACAAA-3′ | R: 5′-ATGGACCATCCTTAGCTTCC-3′ | 219 | [NM_001130211.1](https://www.ncbi.nlm.nih.gov/entrez/viewer.fcgi?db=nucleotide&id=198282000) |
| ***SLC2A1*** | F: 5′-GATGTCCTACCTGAGCATCG-3′ | R: 5′-GCTCCACATACTGGAAGCAC-3′ | 191 | [XM_021096908.1](https://www.ncbi.nlm.nih.gov/entrez/viewer.fcgi?db=nucleotide&id=1191892363) |
| ***SLC2A5*** | F: 5′-AATATCCTGGATGCCCTACC-3′ | R: 5′-CCACTTGAATGAATGGGAAG-3′ | 203 | [XM_021095282.1](https://www.ncbi.nlm.nih.gov/entrez/viewer.fcgi?db=nucleotide&id=1191888310) |
| ***ATP5F1A*** | F: 5′-GCTGCCACTCAACAACTCTT-3′ | R: 5′-TGGCTGATAACGTGAGACAA-3′ | 191 | [NM_001185142.1](https://www.ncbi.nlm.nih.gov/entrez/viewer.fcgi?db=nucleotide&id=297591974) |
| ***GPX1*** | F: 5′-CCTGAATTGCCTCAAGTACG-3′ | R: 5′-AAGTTCCATGCGATGTCATT-3′ | 216 | [NM_214201.1](https://www.ncbi.nlm.nih.gov/entrez/viewer.fcgi?db=nucleotide&id=47523487) |
| ***NRF2*** | F: 5′-CCCATTCACAAAAGACAAACATTC-3′ | R: 5′-GCTTTTGCCCTTAGCTCATCTC-3′ | 75 | XM_021075133 |
| ***KEAP1*** | F: 5′-AGCTGGGATGCCTCAGTGTT-3′ | R: 5′-AGGCAAGTTCTCCCAGACATTC-3′ | 100 | NM_001114671 |
| ***GCLC*** | F: 5′-GTTTTGTGAATCAGGACCCTA-3′ | R: 5′-GCTTAGCTGAAGCTTTATTGC-3′ | 212 | XM_003483635_4 |
| ***GSR*** | F: 5′-TGGGCTCTAAGACGTCACTG-3′ | R: 5′-TCTATGCCAGCATTCTCCAG-3′ | 106 | XM_003483635 |
| ***NQO1*** | F: 5′-ATGAACTTCAATCCCGTCAT-3′ | R: 5′-CTCGGCAGGATACTGWGT-3′ | 191 | NM_0011596 |
| ***SOD1*** | F: 5′-GTGCAGGGCACCATCTACTT-3′ | R: 5′-AGTCACATTGCCCAGGTCTC-3′ | 222 | NM_001190422 |
| ***HO-1*** | F: 5′-AAGGCTTTAAGCTGGTGATG-3′ | R: 5′-GAAGTAGAGGGGCGTGTAGA-3′ | 104 | NM_001004027.1 |
| ***SOD2*** | F: 5′-GACAAATCTGAGCCCTAACG-3′ | R: 5′-GTTAGAACAAGCGGCAATCT-3′ | 193 | NM_214127.2 |

**Supplementary Table 2.** Primer sequences for mitochondrial function and antioxidative genes
